# Control-Informed Reinforcement Learning for Chemical Processes

**DOI:** 10.1021/acs.iecr.4c03233

**Published:** 2025-02-20

**Authors:** Maximilian Bloor, Akhil Ahmed, Niki Kotecha, Mehmet Mercangöz, Calvin Tsay, Ehecatl Antonio del Río-Chanona

**Affiliations:** † Department of Chemical Engineering, Imperial College London, London, South Kensington SW7 2AZ, U.K.; ‡ Department of Computing, Imperial College London, London, South Kensington SW7 2AZ, U.K.

## Abstract

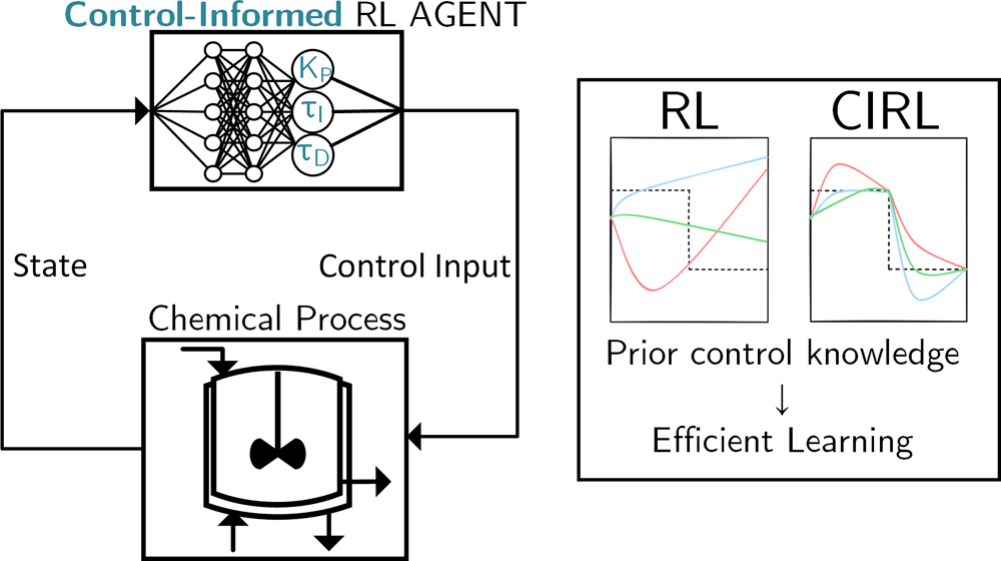

This work proposes a control-informed reinforcement learning (CIRL) framework that integrates proportional-integral-derivative (PID) control components into the architecture of deep reinforcement learning (RL) policies, incorporating prior knowledge from control theory into the learning process. CIRL improves performance and robustness by combining the best of both worlds: the disturbance-rejection and set point-tracking capabilities of PID control and the nonlinear modeling capacity of deep RL. Simulation studies conducted on a continuously stirred tank reactor system demonstrate the improved performance of CIRL compared to both conventional model-free deep RL and static PID controllers. CIRL exhibits better set point-tracking ability, particularly when generalizing to trajectories containing set points outside the training distribution, suggesting enhanced generalization capabilities. Furthermore, the embedded prior control knowledge within the CIRL policy improves its robustness to unobserved system disturbances. The CIRL framework combines the strengths of classical control and reinforcement learning to develop sample-efficient and robust deep reinforcement learning algorithms with potential applications in complex industrial systems.

## 1 Introduction

In the chemical process industry, maintaining control over complex systems is crucial for achieving reliable, efficient, and high-performance operations.^1^ Traditionally, process control has relied heavily on classical feedback control techniques such as proportional-integral-derivative (PID) controllers due to their simplicity, interpretability, and well-established tuning methods.^2^ However, these tuning methods are often largely empirical or otherwise rely on having accurate mathematical models of the open-loop system dynamics and disturbance responses, which can be challenging to derive for complex processes involving nonlinearities, delays, constraints, and changing operating conditions.^3^ Despite decades of development in PID control technology, significant challenges persist. The manual effort required for controller tuning remains substantial, particularly when process conditions change. Furthermore, PID controllers often struggle to provide adequate performance for highly nonlinear and time-varying systems without extensive retuning or gain scheduling.^4^ These limitations have motivated the exploration of more advanced control strategies. One alternative is model predictive control (MPC), a successful model-based process control strategy with the ability to optimize control actions based on the current system states and predicted future behavior while satisfying constraints. This has led to its widespread adoption in the chemical process industry.^5^ Typically, MPC operates as a supervisory layer below the real-time optimization (RTO) layer, providing control set points to lower-level regulatory controllers, often PID controllers, which directly manipulate process variables. This hierarchical structure combines the predictive capabilities of the MPC with the rapid response of traditional feedback control. However, the performance of MPC heavily relies on the accuracy of its internal process model.^6^ The ongoing digitalization of the chemical process industry has opened new avenues for enhancing MPC performance through data-driven approaches. This digital transformation has enabled the integration of advanced analytics and machine learning techniques into both MPC and regulatory control layers, especially on the modeling end. For instance, artificial neural networks^7^ and Gaussian processes^8^ have been employed to capture complex process dynamics that may be challenging to model using purely mechanistic approaches. Furthermore, the increased availability of process data has facilitated the development of hybrid models that combine first-principles knowledge with data-driven components.^9,10^ These advancements, coupled with improvements in computational capabilities, have expanded the applicability of MPC to more complex and uncertain processes. However, challenges remain in areas such as online computational requirements for large-scale systems and the handling of uncertainties.

Recently, reinforcement learning (RL) has emerged as a promising data-driven framework for learning control policies directly from interactions with the chemical process system.^11^ Deep RL methods, which utilize deep neural networks (DNNs), have demonstrated success in a variety of difficult decision-making and control problems. A key advantage of model-free RL is that it does not require accurate system models once online, instead learning control policies from experience. While model-free RL approaches can learn control policies without requiring explicit system models, they often face challenges in sample efficiency and may not fully leverage existing domain knowledge.^12^

For safety reasons, RL algorithms in chemical process control are typically trained on simulation models rather than directly on physical systems. Despite this limitation, the RL offers several advantages over traditional control methods. One significant benefit is the fast online inference time. This characteristic makes RL particularly suitable for systems where online computation time is critical, as the trained policy can execute control decisions rapidly in real-time applications. RL also shows promise in handling complex, nonlinear systems and adapting them to process uncertainties. This feature allows the RL to potentially address challenges in dynamic chemical processes more effectively than traditional control approaches. We note there is also growing interest in algorithms for *safe* RL or those that can avoid (known or unknown) constraints, e.g., in physical systems.^13^

It is important to acknowledge the challenges associated with implementing RL to control complex chemical processes. The offline training of RL agents often requires a large number of samples to achieve satisfactory performance, making the training process computationally intensive and time-consuming. The quality of the trained agent is highly dependent on the fidelity of the simulation model used, which may not always capture all of the nuances of real-world processes, requiring online fine-tuning. Moreover, deep RL methods often treat the control problem as a black box, failing to incorporate valuable insights from the control theory. This highlights the need for approaches that can balance the model-free learning capabilities of RL with the incorporation of domain expertise and efficient exploration strategies.

### 1.1 Related Works

Deep reinforcement learning (deep RL), which combines DNNs with RL, has been demonstrated in various domains, including robotics, data center operations, and playing board games.^14−16^ This success has brought attention to RL from the process and control communities. The process systems engineering community has made significant progress in adapting RL to the process industries, including in distributed systems,^17^ constraint handling,^18−20^ inventory management,^18,21^ batch bioprocess control,^22−24^ production scheduling,^25^ and energy systems.^26^ Early applications of RL in process control proposed model-free RL for tracking control and optimization in fed-batch bioreactors.^27−29^ More recently, Mowbray et al. employed a two-stage strategy using historical process data to warm-start the RL algorithm and demonstrated this on three set point-tracking case studies.^30^ Machalek et al. developed an implicit hybrid machine learning model combining physics-based equations with artificial neural networks, demonstrating its application for reinforcement learning in chemical process optimization.^31^ Zhu et al. developed an RL algorithm that improves scalability by reducing the size of the action space, which was demonstrated on a plantwide control problem.^32^ However, the sample efficiency of these algorithms remains a key aspect restricting their widespread industrial adoption.

To address the limitations, prior works have explored integrating reinforcement learning with existing control structures, e.g., PID controllers.^33−35^ Early approaches applied model-free RL to directly tune the gains of PID controllers^36,37^ or used model-based RL techniques such as dual heuristic dynamic programming.^38^ Other approaches have investigated embedding knowledge of the dynamical system using physics-informed neural networks to act as a surrogate model of the process for offline training of the RL agent.^39^ Efforts have also been made to develop interpretable control structures that maintain transparency while leveraging advanced optimization techniques.^40^ Lawrence et al. directly parametrized the RL policy as a PID controller instead of using a DNN, allowing the RL agent to improve the controller’s performance while leveraging existing PID control hardware.^41^ This work demonstrates that industries can utilize actor-critic RL algorithms without the need for additional hardware or the lack of interpretability that often accompanies the use of a DNN. To improve this work’s training time, McClement et al. used a meta-RL approach to tune PI controllers offline.^42^ The method aimed to learn a generalized RL agent on a distribution of first-order plus time delay (FOPTD) systems, resulting in an adaptive controller that can be deployed on new systems without any additional training. However, while the meta-RL approach removes the need for explicit system identification, some knowledge of the process gain and time constant magnitudes is still required to appropriately scale the meta-RL agent’s inputs and outputs when applying it to new systems.

The time-invariant PID structure can achieve good performance on approximately linear systems. Historically, this condition was often sufficient, as processes are often operated around a known set point in an approximately linear region; however, more recent applications in control of nonlinear systems (e.g., transient, intensified, or cyclic processes) motivate more advanced control strategies. Given a nonlinear system, the selection of effective PID parameters will be dependent on the operating point, which motivates a gain-scheduled approach. Gain scheduling involves designing multiple PID controllers for different operating regions and switching between them based on the current process conditions. In industrial applications, a common approach to gain scheduling is through the use of lookup tables, where the PID gains are precomputed and stored for different operating conditions or set points.^43^ More recently, data-driven, model-free approaches to gain scheduling have gained traction as they can potentially design the control strategy directly from a single set of plant input and output data without the need for system identification.^44^ Despite the advancements in PID control, challenges remain in terms of the manual effort required for controller tuning and the limited performance in highly nonlinear and time-varying systems. These challenges motivate the integration of PID control with data-driven and learning-based approaches, such as reinforcement learning, to leverage the strengths of both paradigms.

### 1.2 Contributions

While previous works have explored using reinforcement learning to tune static PID gains or implementing gain scheduling strategies, we propose a control-informed reinforcement learning (CIRL) framework that embeds PID control structures directly within the architecture of deep RL policies. Unlike methods that treat gain tuning as a separate optimization problem, our approach enables the continuous adaptation of PID gains through an integrated neural network, allowing the controller to respond to changing operating conditions in real time. By unifying PID control and deep RL in a single architecture, CIRL leverages the proven stability and interpretability of PID control while harnessing deep RL’s capacity for nonlinear modeling and adaptation:

1. We introduce the CIRL framework, which augments deep RL policies with an embedded PID controller layer. This enables the agent to learn adaptive PID gain tuning while preserving the stabilizing properties and interpretability of PID control, effectively acting as an automated gain scheduler.
2. We demonstrate the CIRL framework on a nonlinear continuously stirred tank reactor (CSTR) system. The CIRL agent improves set point tracking performance compared to both a static PID controller and a standard model-free deep RL approach, showing robust performance even with moderate deviations from training set points.
3. We show that by leveraging the embedded prior knowledge from the PID structure, the CIRL agent exhibits enhanced robustness to process disturbances that are not observable during training.

The remainder of this article is organized as follows: Section 2 presents the background on PID control and reinforcement learning. Section 3 describes the proposed CIRL framework in detail. Section 4 discusses the simulation and experimental results. Finally, Section 5 concludes the article and outlines future research directions.

## 2 Background

### 2.1 Reinforcement Learning

**Figure 1:**
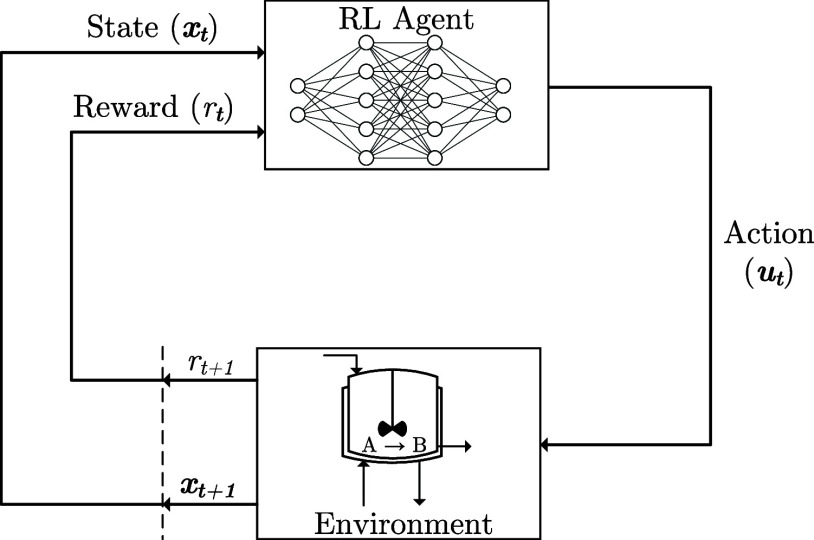
RL framework.

The standard RL framework (Figure 1) consists of an agent that interacts with an environment. Assuming the states are fully observable, the agent receives a vector of measured states  and can then take some action , which results in the environment progressing to state **x**_*t*+1_. Sets  and  represent the state and action space, respectively. For a deterministic policy π, the agent takes actions **u**_*t*_ = π(**x**_*t*_), while for a stochastic policy, the action **u**_*t*_ is sampled from the policy π represented by a conditional probability distribution **u**_*t*_ ∼ π(·| **x**_*t*_). A common assumption in RL is that the state transition given some action is defined by the density function ***x***_*t*+1_ ∼ *p*(·| **x**_*t*_, **u**_*t*_) that represents the stochastic nonlinear dynamics of the process. The reward the agent receives is defined by the function . With a defined control policy, the policy can be implemented over a discrete time horizon *T* thus producing the following trajectory τ = (**x**_0_, **u**_0_, *r*_0_, **x**_1_, **u**_1_, *r*_1_,..., **x**_*T*_, **u**_*T*_, *r*_*T*_).

**Figure 2:**
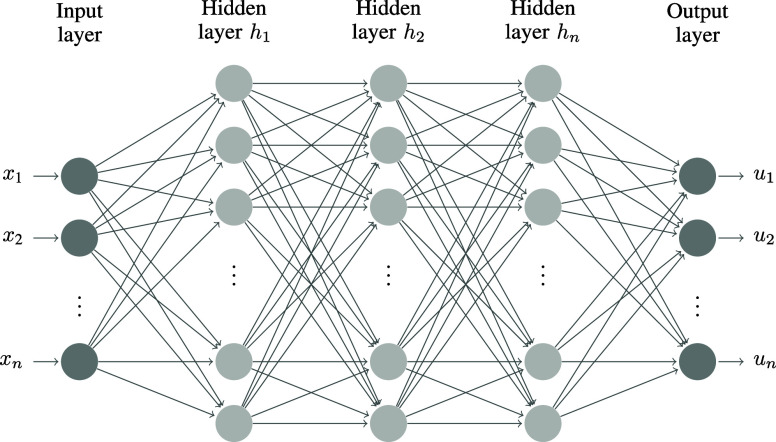
Deep policy network π_θ_.

Formally, notice that the above state transition assumption enables modeling the underlying system as a Markov decision process (MDP); for further treatment of the subject, the reader is referred to Sutton and Barto.^45^ In reinforcement learning, particularly in our framework, the agent’s goal is to maximize the cumulative reward (return) *J*(π) over a predefined (often infinite) timespan, and a discount factor γ is used to reflect the uncertain future and ensure computational tractability. The policy that achieves this is the optimal policy π*:
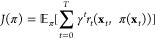
1
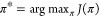
2To address the challenges of continuous state and action spaces in industrial control problems, DNNs are commonly used as function approximators for the policy. This allows parametrization of the policy π ≈ π_θ_, where θ ∈ Ω ⊆  represents the neural network parameters and Ω is the parameter space (Figure 2). Various approaches exist for optimizing these policy parameters to maximize the expected return. Popular policy gradient methods such as trust region policy optimization (TRPO)^46^ and proximal policy optimization (PPO)^47^ use gradient-based updates to improve the policy parameters directly. These methods have shown success in continuous control tasks but can be sensitive to hyperparameter choices and require careful implementation to ensure stable learning. In this work, we instead employ evolutionary strategies as detailed in Section 2.2, which offer advantages in terms of parallelization and exploration compared to gradient-based methods. The evolutionary approach also aligns well with our control-informed architecture, as it does not require differentiation through the PID control layer. Both policy gradient methods and evolutionary strategies are viable approaches depending on the specific problem requirements with our evolutionary strategy implementation serving as an effective initial demonstration.

### 2.2 Evolutionary Strategies in Reinforcement Learning

Within policy optimization methods, in addition to algorithms that leverage policy gradients, it is also possible to use evolutionary algorithms. Both types of algorithms update the parameters to optimize the policy, which takes states as inputs and outputs (optimal) control actions (Figure 2). This distinction is not unlike evolutionary and gradient-based algorithms in traditional optimization problems, i.e., evolutionary algorithms simply provide an alternative framework for learning the policy parameters. Evolutionary strategies (ES) are a class of data-driven optimization algorithms inspired by principles of biological evolution. These algorithms optimize policies by iteratively generating populations of candidate solutions, evaluating their fitness (performance), and selectively propagating the fittest individuals to subsequent generations through processes similar to mutation, recombination, and selection.

In the context of RL, ES can be used to optimize parameters **θ** of a policy π_**θ**_ directly, without relying on gradient information. These policies are evaluated in the environment on an episodic basis with their cumulative return as shown by a parametrized variant of eq 1:
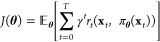
3Instead of using the approximation of the *Q*-function, we use a return gradient estimate to optimize the policy parameters. ES-RL algorithms use the estimate of the *J*(**θ**) and directly update the parameters toward those policies that produce higher returns to improve performance.^48^ While evolutionary strategies (ES) represent one approach to training RL policies, they have distinct trade-offs compared to policy gradient methods. ES methods can be advantageous when working with hybrid architectures such as CIRL, as they treat the combined PID-neural network system as a black box without requiring backpropagation through the control structure. They are also less susceptible to getting trapped in local optima compared to gradient-based methods as they explore the parameter space more “globally” through population-based search. Nevertheless, ES approaches face important limitations, particularly in scaling to larger neural networks due to the curse of dimensionality in population-based optimization. For the specific case of CIRL, where the PID structure effectively handles a large part of the control complexity, we found that ES provided stable training with small networks (16 neurons per layer). This aligns with recent applications of ES-RL showing success in specific domains. Both ES and policy gradient methods are valid approaches, depending on the specific problem requirements and constraints. Salimans et al. applied and developed an ES-RL algorithm and evaluated it on MuJoCo and Atari environments resulting in comparable performance to policy gradient methods such as TRPO.^46,49^ Wu et al. used a hybrid strategy of derivative-free optimization techniques to solve an inventory management problem with improved performance over the policy gradient method PPO.^47,50^

### 2.3 PID Controllers

The PID controller is a widely used feedback mechanism employed in industrial control systems.^3^ The discrete PID controller calculates an error value *e*_*t*_ in discrete time as the difference between a desired set point and a measured process variable and applies a correction based on three parameters: proportional (*K*_p_), integral time constant (τ_i_), and derivative time constant (τ_d_). Note that other parametrizations of these degrees of freedom are possible. The proportional term applies a control action proportional to the current error, providing an immediate response to deviations from the set point. The integral term accumulates the error over time and applies a control action to eliminate steady-state errors. The derivative term considers the rate of change of the error and provides a dampening effect to prevent overshoots and oscillations. The discrete position form of a single PID controller is defined as
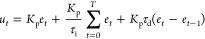
4where *e*_*t*_ = *x*_*i*,t_^*^ – *x*_*i*,*t*_ is the set point error of state *i* at timestep *t*, with the set point *x*_*i*,t_^*^ of state *i* at timestep *t*.

Tuning the PID gains refers to finding values of the parameters *K*_p_, τ_i_, and τ_d_ that result in good closed-loop performance (often measured by integrated squared error, etc.) and is crucial for achieving desired control performance. As a result, many popular tuning methodologies have been developed, including the internal model control^51,52^ and relay tuning.^53^ The first of these methods is a model-based technique, and the second excites the system and uses the response to estimate the three PID parameters.

## 3 Methodology

This section presents the proposed CIRL framework, which integrates PID control structures into the policy architecture of deep RL agents. The methodology covers the CIRL agent design, policy optimization algorithm, and implementation details.

### 3.1 CIRL Agent

**Figure 3:**
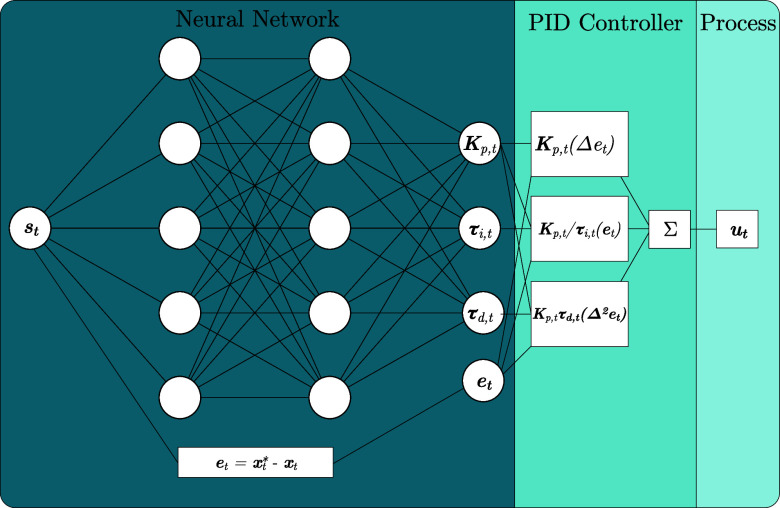
CIRL agent.

The CIRL agent consists of a DNN policy augmented with a PID controller layer, as illustrated in Figure 3. The base neural network takes the observed states **s**_*t*_ as inputs and outputs the PID gain parameters *K*_p,*t*_, τ_i,*t*_, and  at each timestep *t*. The PID controller layer then computes the control action **u**_*t*_ based on the error signal e_*t*_ = **x**_t_^*^ – **y**_*t*_ and the current learned gain parameters where **y**_*t*_ is the measurement of the system’s internal state at time *t*.

The agent’s state **s**_*t*_ ∈ *S* ⊆ *R*^*n*_s_^, which consists of current and historical measurements ***y***_*t*...*t*–*N*_*t*__ derived from the system’s internal state **x**_*t*_, includes *N*_*t*_ timesteps of history for both the state and set point, where *N*_*t*_ > 2 to fully define the velocity-form PID controller:
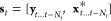
5The PID layer is represented in the velocity form since, if the position form of the PID controller (eq 4) is used and the gain changes suddenly, this can cause disturbances to the system.^4^ The velocity form mitigates this issue by ensuring that the control input does not change abruptly, despite sudden gain changes, and it is not necessary to reset the integral term. The *k*^th^ PID controller of the system is represented by
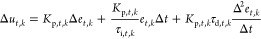
6where Δ*e*_*t*,*k*_ = *e*_*t*,*k*_ – *e*_*t*–1,*k*_, Δ^2^*e*_*t*,*k*_ = Δ*e*_*t*,*k*_ – 2*e*_*t*–1,*k*_ + *e*_*t*–2,*k*_, and the subscript (*k*) denotes the index of the controller, where *k* ∈ 0, 1,..., *n*_*u*_, and *n*_*u*_ is the total number of controllers in the system.

Through interacting with the environment, the CIRL agent aims to maximize the cumulative reward given by *r*_*t*_ ∈  at each timestep. For process control regulatory problems, various reward functions have been proposed. In general, they all involve some measure of (integrated) set point error, either squared or absolute, and/or a penalty for control action, similar to MPC objective functions. Adopting a similar notation to MPC, a squared error term penalizes deviations of the controlled variable from the set point, with larger deviations penalized more heavily:
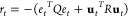
7where ***Q*** ∈ ^*n*_*x*_ × *n*_*x*_^ and ***R*** ∈ ^*n*_*u*_ × *n*_*u*_^ are weighting factors that balance the trade-off between tracking performance and control effort.

It is important to note that derivative information is not passed between the PID controller and the neural network in the proposed CIRL agent architecture, as we use an evolutionary optimization strategy. Future work may study an integrated gradient-based learning strategy.

### 3.2 Policy Optimization Method

In this work, the CIRL agent’s policy is optimized using a hybrid approach based on evolutionary strategies, combining random search and particle swarm optimization (PSO).^54^ A population of candidate policy parameter vectors is initialized by sampling randomly from the allowable ranges for each parameter dimension. The parameters in this case are the weights of the neural networks. This initial random sample provides a scattered set of starting points that encourage exploration of the full parameter landscape. The random population undergoes *N* iterations in which the objective function value (cumulative reward obtained by the policy in the environment) is evaluated for each policy to initialize the population in a good region of the policy space. The objective function to be maximized is
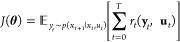
8The expectation  is taken over the system dynamics, capturing how states evolve according to the transition probability *p*(*x*_*t*+1_|*x*_*t*_, *u*_*t*_). While the policy π_**θ**_ is deterministic, the state transitions may remain stochastic due to process/measurement noise. The expectation therefore averages the reward over all possible state trajectories that could occur under this uncertainty. The best, or *fittest*, policies from this initial random sampling are carried forward as seeds to initialize the PSO phase of the algorithm. The PSO phase is then started, allowing the particles (policy parameter vectors **θ**_*i*_) to explore areas around the initially fit random vectors in a more structured manner. In each PSO iteration, particle velocities and positions are updated as
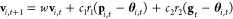
9
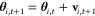
10where **v**_*i*,*t*_ and **θ**_*i*,*t*_ are the velocity and policy parameter vector of particle *i* at iteration *t*, *w* is the inertia weight, *c*_1_ and *c*_2_ are the cognitive and social acceleration constants, *r*_1_ and *r*_2_ are random numbers in [0, 1], **p**_*i*,*t*_ is the personal best policy parameter vector of particle *i*, and **g**_*t*_ is the global best policy parameter vector at iteration *t*. This hybrid approach leverages the global exploration capabilities of initial random sampling while also taking advantage of the PSO’s ability to collaboratively focus its search around promising areas identified by the initial random search. The pseudocode for the policy optimization procedure is given by Algorithm 1. We highlight that the CIRL framework is agnostic to the policy optimization strategy; i.e., policy gradients or other policy optimization techniques can be used. Our choice of evolutionary algorithms in this work is motivated by optimization performance given the small size of the neural network as well as robustness in training.

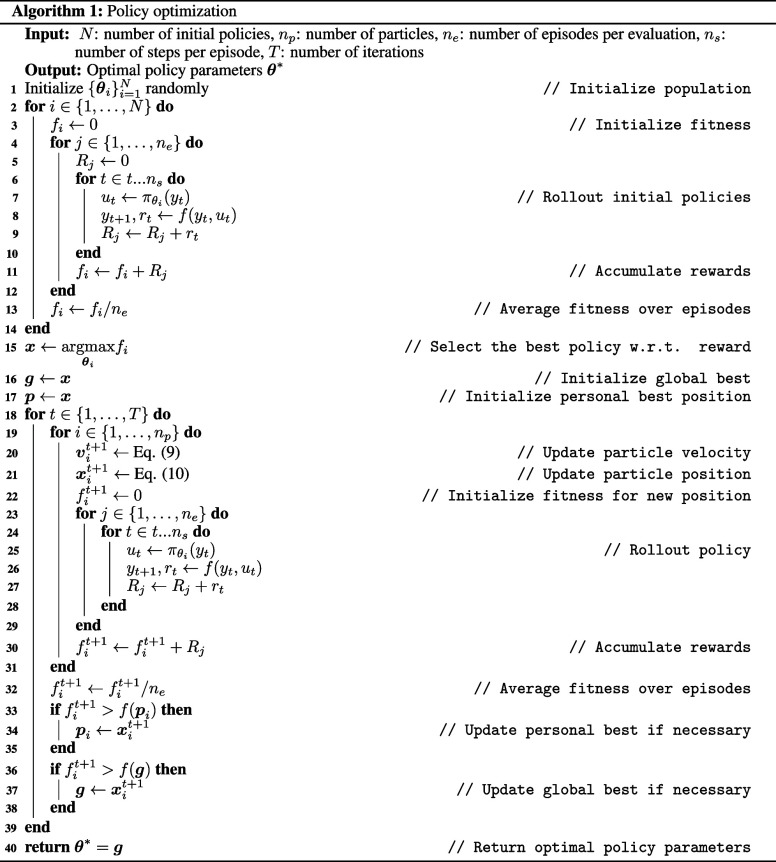



## 4 Results and Analysis

### 4.1 Computational Implementation

This section outlines the computational implementation employed in our study. We describe the state representation, neural network architecture, optimization parameters, and benchmark comparisons used to evaluate our proposed approach. Additionally, we provide information about the computational resources used. The RL state *s*_*t*_ representation used for the CIRL agent included *N*_*t*_ = 2 timesteps of history, which is the minimum number of timesteps to define the PID controller layer:
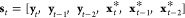
11The neural network architecture of the CIRL agent consists of three fully connected layers, each containing 16 neurons with ReLU activation functions, with the output being clamped to the normalized PID gain bounds. The CIRL agent is compared to a deep RL approach (without the PID control structure, which we refer to as pure-RL) and is also optimized using evolutionary strategies. This pure-RL agent consists solely of a DNN without the PID layer; we found this to require a larger network size and use three fully connected layers with 128 neurons. While other architectures incorporating previous information, such as recurrent neural networks (e.g., LSTMs and GRUs), could be employed, we opted for this simpler structure in the present study. For the PSO algorithm used for policy optimization, we define the inertia weight *w* as 0.6, while both the cognitive and social acceleration constants (*c*_1_ and *c*_2_, respectively) are set to 1. The policy optimization algorithm is initialized with *N* = 30 policies, before starting the PSO loop for *T* = 150 iterations with *n*_p_ = 15 particles. In this PSO loop, *n*_e_ = 3 episodes are used for each policy evaluation, with *n*_s_ = 120 timesteps in each rollout. All training was conducted on a 64-bit Windows laptop with an Intel i7-1355U CPU at 3.7 GHz and an NVIDIA RTX A500 (Laptop) GPU. The CIRL agent required approximately 10 min of training time.

### 4.2 CSTR Case Study

**Figure 4:**
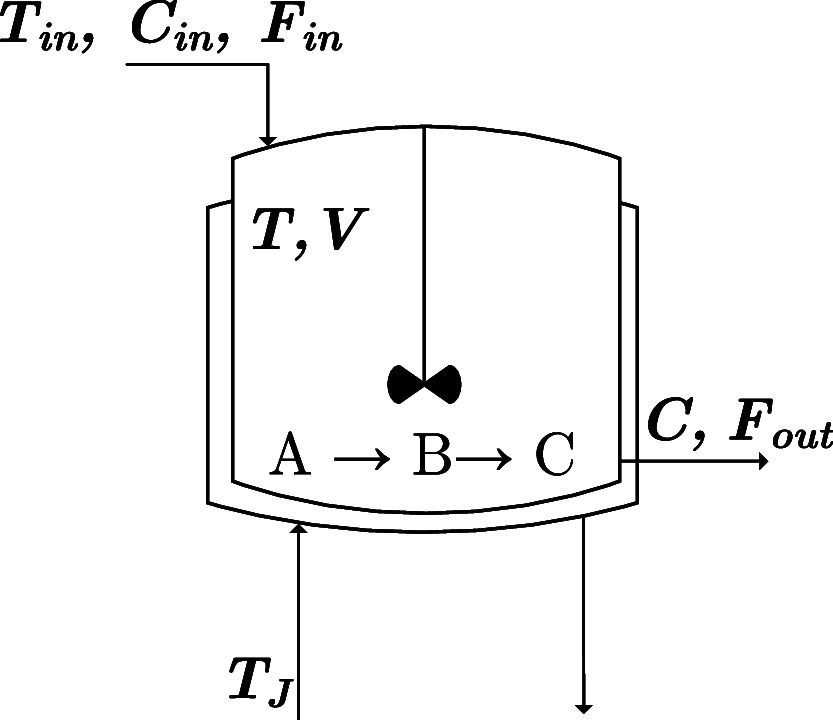
CSTR process flow diagram.

To demonstrate the proposed algorithm, simulation-based experiments were carried out on a CSTR system (Figure 4) where both the volume and temperature are controlled, which is adapted from refs (3) and (55). Though conceptually simple, this case study represents a nontrivial, multivariable system with nonlinear dynamics, capturing many challenges representative of those in real-world processes.

The following generalized reactions take place in the reactor where B is the desired component:
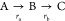
12The following system of ordinary differential equations models the dynamics of the three chemical components in the reactor: *C*_A_ (concentration of A in mol/m^3^), *C*_B_ (concentration of B in mol/m^3^), and *C*_C_ (concentration of C in mol/m^3^), respectively.
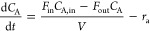
13
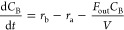
14
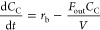
15where *F*_in_ is the volumetric flow of feed into the system (m^3^/min), *C*_A,in_ is the feed concentration of species A (mol/m^3^), *r*_j_ is the reaction rate for reaction j (mol/m^3^/min), and *V* is the volume of the CSTR (m^3^). For this subsystem to be fully defined, the reaction rates are described by Arrhenius relationships for both reactions:
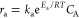
16
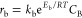
17where *k*_a_ and *k*_b_ are the Arrhenius rate constants (s^–1^), *E*_A_ and *E*_B_ are the activation energies (J/mol), *R* is the universal gas constant (8.314 J/mol K), and *T* is the temperature (K). The dynamics of the reactor temperature *T* (K) and volume *V* (m^3^) are described by the following ordinary differential equations:
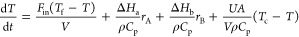
18
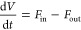
19where *T*_f_ is the inlet stream temperature (K), Δ*H*_A_ and Δ*H*_B_ are the heats of reaction (J/mol), ρ is the density of the solvent (kg/L), *C*_p_ is the heat capacity (J/kg/K), *U* is the overall heat transfer coefficient (J/min/m^2^/K), *A* is the heat transfer area (m^2^), and *T*_c_ is the coolant temperature (K). The parameters used in the simulation experiments are listed in Table 1. The case study was implemented as a gym environment^56^ to provide a standardized format designed for RL algorithms. Within the gym environment, the system of the ODEs is integrated using SciPy’s ODEInt method.

**Table 1:** Parameters for the CSTR Dynamic Model

parameter	value
*T*_f_	350 K
*C*_A,in_	1 mol/m^3^
*F*_out_	100 m^3^/sec
ρ	1000 kg/m^3^
*C*_p_	0.239 J/kg-K
*UA*	5 × 10^4^ W/K
Δ*H*_a_	5 × 10^3^ J/mol
*E*_a_/*R*	8750 K
*k*_a_	7.2 × 10^10^ s^–1^
Δ*H*_b_	4 × 10^3^ J/mol
*E*_b_/*R*	10750 K
*k*_b_	8.2 × 10^10^ s^–1^

The three observed states of the reactor are the concentration of B *C*_B_, reactor temperature *T*, and volume *V*, which define the state vector **x** = [*C*_B_, *T*, *V* ]. We desire a policy that maps these to the action space, comprising the cooling jacket temperature *T*_c_ and the inlet flow rate *F*_in_, defining the control vector **u** = [*T*_j_, *F*_in_]. This creates a system with two PID controllers, the first pairs *T*_c_ and *C*_B_ and the second pairs *F*_in_ and *V*. The pairing was decided using a relative gain array (RGA), which is shown in the appendix. This is additive measurement noise on all states of the CSTR. The system is simulated for 25 min with 120 timesteps. The bounds on the two control inputs are as follows: *u*^L^ = [290 K, 99 m^3^/min] and *u*^U^ = [450 K, 105 m^3^/min]. There are also bounds on the PID gains outputted by the DNN in the CIRL agent, which are given in Table 2. The initial state is defined as *x*_0_ = [0 mol/m^3^, 327 K, and 102 m^3^].

**Table 2:** Bounds on PID Gains

	*C*_B_-loop	*V*-loop
*K*_p_	[−5, 25] (K · m^3^/mol)	[0, 1] (s^–1^)
τ_i_	[0, 20] (s)	[0, 2] (s)
τ_d_	[0, 10] (s)	[0, 1] (s)

### 4.3 Training

The CIRL and pure-RL algorithms were trained on nine set points that span the operating space of the CSTR case study (Figure 9). The operating space is defined for *C*_B_ between 0.1 and 0.8 mol/m^3^ while maintaining a constant volume of 100 *m*^3^. This is with the aim to learn a generalized control policy for a wide range of *C*_B_ set points. Practically, this was achieved by rolling out the policy on the three subepisodes (1–3 in Table 3) and then summing them to create a single reward signal.

**Table 3:** Training and Test Scenarios

subepisode	set point schedule	
	*C*_B_ [mol/m^3^]	*V* [m^3^]
1	0.1 → 0.2 → 0.3	100
2	0.4 → 0.5 → 0.6	100
3	0.7 → 0.75 → 0.86	100
test	0.075 → 0.45 → 0.75	100

**Figure 5:**
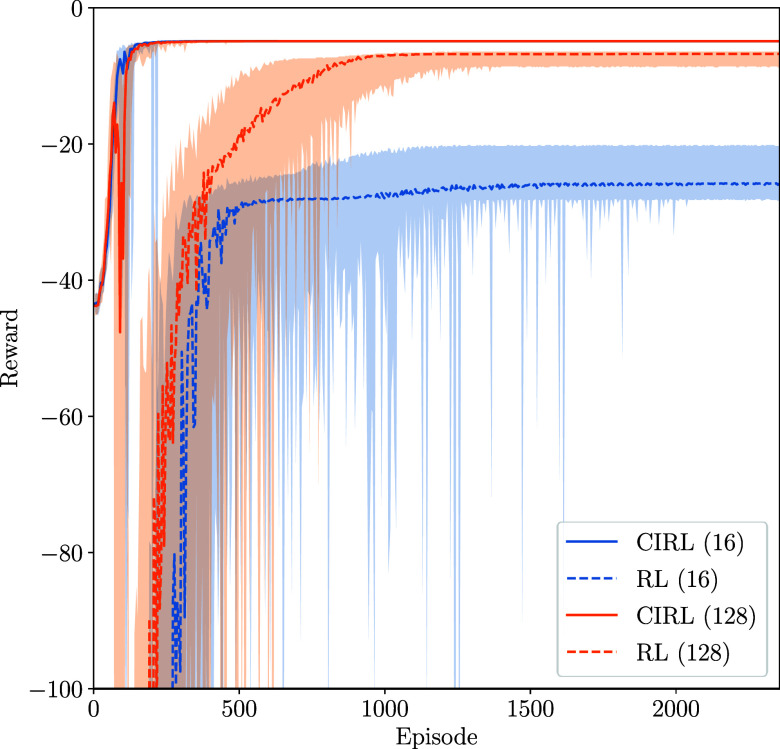
Learning curves for both RL and CIRL policies with 16 and 128 neurons per fully connected layer.

The training regime consists of three subepisodes that span different regions of the operating space, organized by increasing concentration. The second subepisode covers the lower range (0.1 → 0.2 → 0.3 mol/m^3^), followed by the third subepisode in the linear middle range (0.4 → 0.5 → 0.6 mol/m^3^). The first subepisode explores the upper concentration region (0.7 → 0.75 → 0.86 mol/m^3^) where the reaction kinetics become highly nonlinear due to the increased consumption of species B. Each set point is maintained for one-third of the episode length. This structured approach ensures the agent learns to control the system across the full spectrum of concentrations while maintaining sufficient time at each set point. As mentioned above, we found that without the PID layer, a larger DNN policy was required to reach comparable performance. Therefore, the pure-RL algorithm implemented with a larger number of neurons (128) in each fully connected layer still reaches comparable training performance to CIRL (Figure 5).

**Figure 6:**
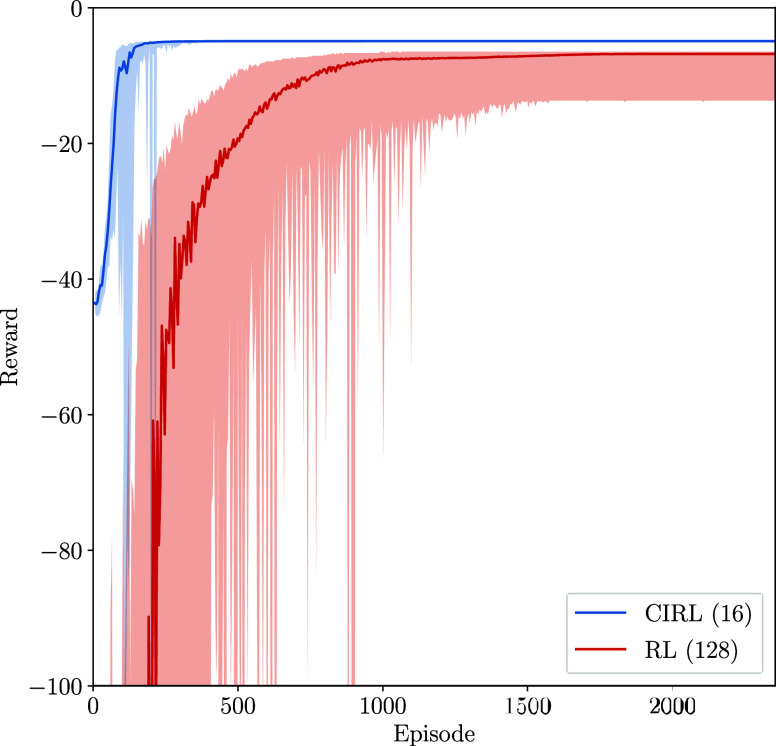
Set point tracking learning curves of CIRL and RL on 10 different seeds. Initial random search is omitted.

The sample efficiency of RL algorithms is one of the main concerns regarding their implementation. Here, we demonstrate the improved sample efficiency of CIRL compared with an RL algorithm with the PID controller removed. The CIRL agent can be seen to initialize at a higher reward than the pure-RL approach since it has prior knowledge of the control strategy and benefits from the inherent stabilizing properties. Furthermore, CIRL exhibits significantly lower variance in reward during training compared to pure-RL approaches (Figure 6). This enhanced stability comes from the structured PID control layer, which provides consistent baseline performance while the neural network component learns to gain adjustments. This leads to more efficient and faster learning compared to pure-RL approaches, as the agent can make informed decisions and requires fewer samples to learn a good policy. In the real world, this corresponds to fewer simulations and experiments before an adequate control policy is obtained. Furthermore, given the stabilizing properties of the PID layer, this results in a safer policy, which inherently maintains set point tracking by utilizing the set point error. The PID controller’s ability to continuously adjust based on the error between the desired set point and the current state provides a fundamental safety mechanism. This makes the overall policy more robust and less prone to dangerous deviations, especially during the early stages of learning, when the neural network component might produce unreliable outputs.

The pure-RL agent, without any prior domain knowledge, must explore a larger number of samples, leading to slower convergence. This agent must learn the control strategy from scratch, including error correction and set point tracking which are inherently built into the CIRL approach. As a result, the pure-RL agent typically exhibits a higher variance in its actions during the early stages of training, as it explores a wider range of potentially suboptimal strategies. The lack of a PID layer means that the neural network in the pure-RL approach is learning to output controls, which is inherently a larger space than the PID-gain space. This often necessitates a larger network architecture, as seen in our implementation with 128 neurons per layer, to capture the complexity of the control task. The increased network size, while providing more expressive power, also increases the dimensionality of the parameter space that must be optimized, potentially leading to longer training times and increased computational requirements. The learning curves over 75 iterations of the policy optimization algorithm for both the CIRL and pure-RL algorithms are shown in Figure 6.

### 4.4 Set Point Tracking: Normal Operation

**Figure 7:**
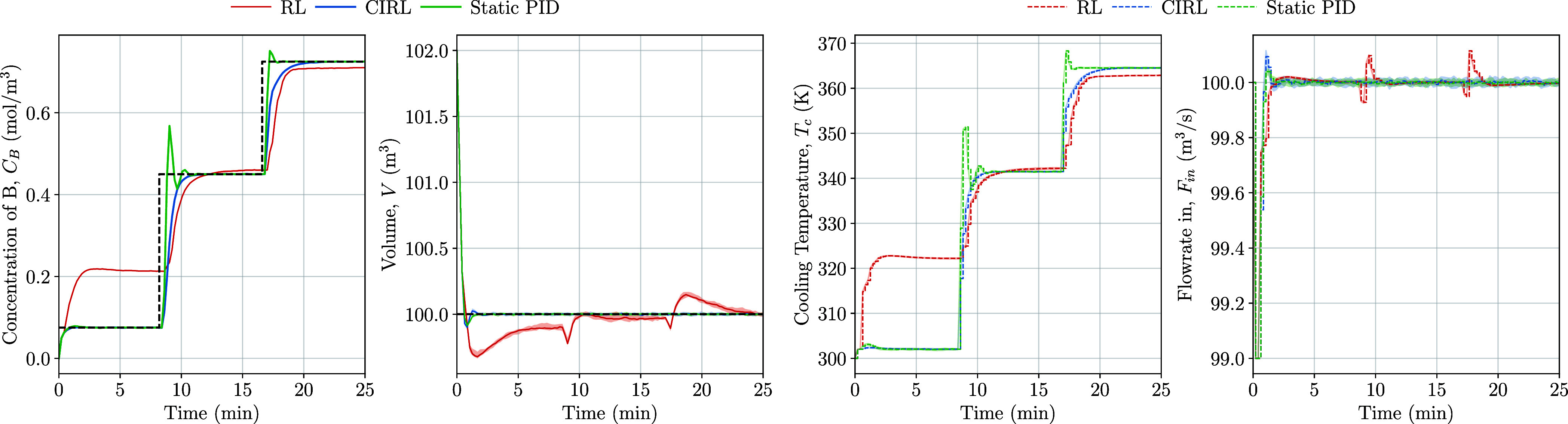
Set point tracking test scenario states and control inputs for CIRL, pure-RL, and static PID.

We then test both learned policies on a partially unseen set point-tracking task. Specifically, the trained policies are then tested on a set point schedule detailed in Table 3, which consists of three set points: the first set point is outside the training regime (shown in bold), and the other two interpolate between the training set points. The CIRL agent is compared to the pure-RL agent described previously in Section 4.1 and a static PID controller. The static PID controller was tuned with a differential evolution strategy to find gains using the set points in the training regime (Table 3). The gains found for the static PID controller are listed in Table 4. Then these three controllers were simulated on the test scenario (Table 3) and are shown in Figure 7.

**Table 4:** PID Gains for the Static PID Controller

	*C*_B_-loop	*F*_in_-loop
*K*_p_	3.09 K · m^3^/mol	0.84 s^–1^
τ_*i*_	0.03 s	1.85 s
τ_d_	0.83 s	0.08 s

**Figure 8:**
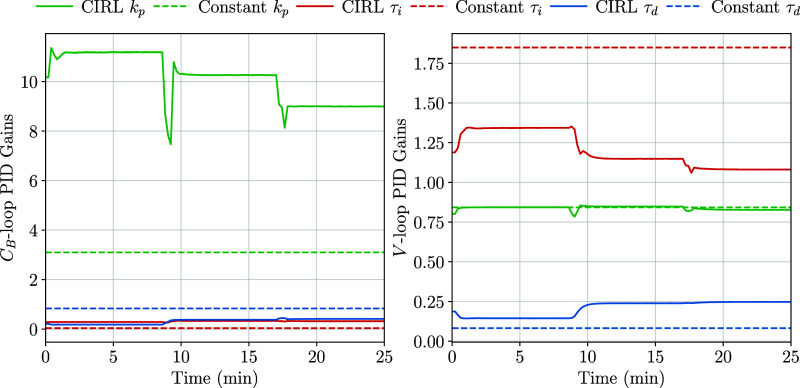
Gain trajectories for the *C*_B_ and *V* loop controllers.

The pure-RL approach in Figure 7 exhibited poor tracking when generalizing to the out-of-distribution set point (*x*_*C*_B__* = 0.075 mol/m^3^) shown by the larger lower test reward (Table 5). By manipulating the proportional, integral, and derivative terms of its internal PID controller (Figure 8), the CIRL policy could adapt its control outputs to track previously unseen set point trajectories. While CIRL demonstrates stability when handling the previously unseen set point of 0.075 mol/m^3^, which lies below the training regime’s lower bound of 0.1 mol/m^3^, we expect its performance may degrade for set points far from the training distribution. The improved stability compared to pure-RL in this intermediate region likely stems from the embedded PID structure, providing basic regulatory control even under unfamiliar conditions. This ability to adaptively tune the PID gains allowed CIRL to outperform not only the model-free RL baseline approach but also a static PID controller tuned to the set points in the training data. The static PID controller gains were optimized using differential evolution,^57^ a population-based optimization algorithm well-suited for nonconvex optimization problems. The optimization objective was to maximize the cumulative reward (minimize tracking error and control effort) over all set points in the training regime (Table 7). The differential evolution algorithm was configured to explore all six PID parameters (proportional, integral, and derivative gains for both loops). The optimization used a population size proportional to the parameter dimension and ran for 150 iterations to ensure convergence. Each candidate solution was evaluated by simulating the closed-loop system response across all training set points to find gains that provide good overall performance across the operating range. These results highlight the key benefit of the CIRL approach: integrating interpretable control structures like PID into deep RL enables performance gains compared with either component in isolation.

**Table 5:** Final Test Reward for Pure-RL, CIRL, and Static PID (Best Reward Shown in Bold)under the Normal Operation Scenario

method	test reward
RL	–2.08
**CIRL**	**–1.33**
static PID	–1.77

### 4.5 Set Point Tracking: High Operating Point

**Figure 9:**
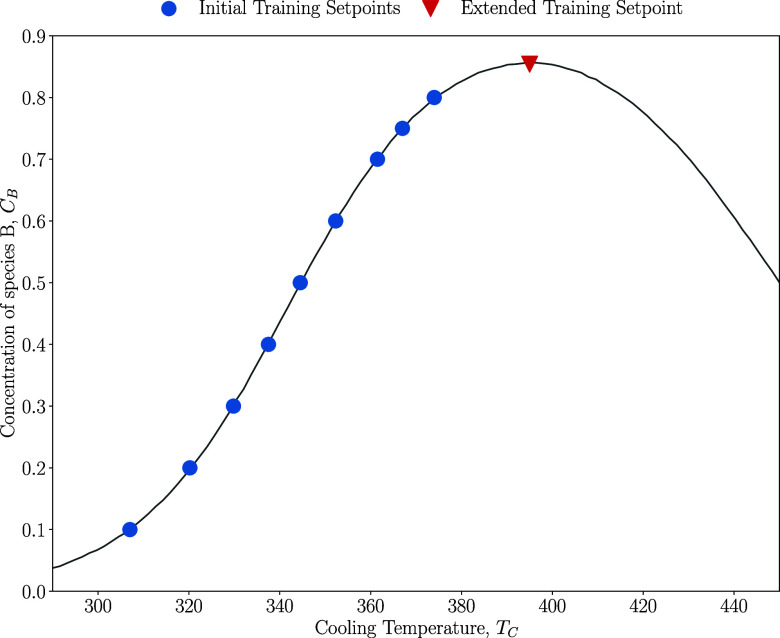
Operating space at a fixed *V* = 100 m^3^ with initial and extended training set points.

The CIRL algorithm does outperform the static controller in normal operation; however, the benefits are marginal and could potentially be attributed to an overtuned controller. We now consider a more challenging operating scenario: if the operating point is pushed to a region of the operating space (red triangle in Figure 9), the gradient changes sign, as can be seen at cooling temperatures above 390 K. This is due to the second reaction rate increasing and consuming species B, causing the relationship between cooling temperature and concentration to become negative rather than positive. This also poses a problem to the PID controller and PID layer in CIRL since to maximize the concentration of species B, the proportional gain must decrease and potentially change sign to stabilize around the maximum.

**Figure 10:**
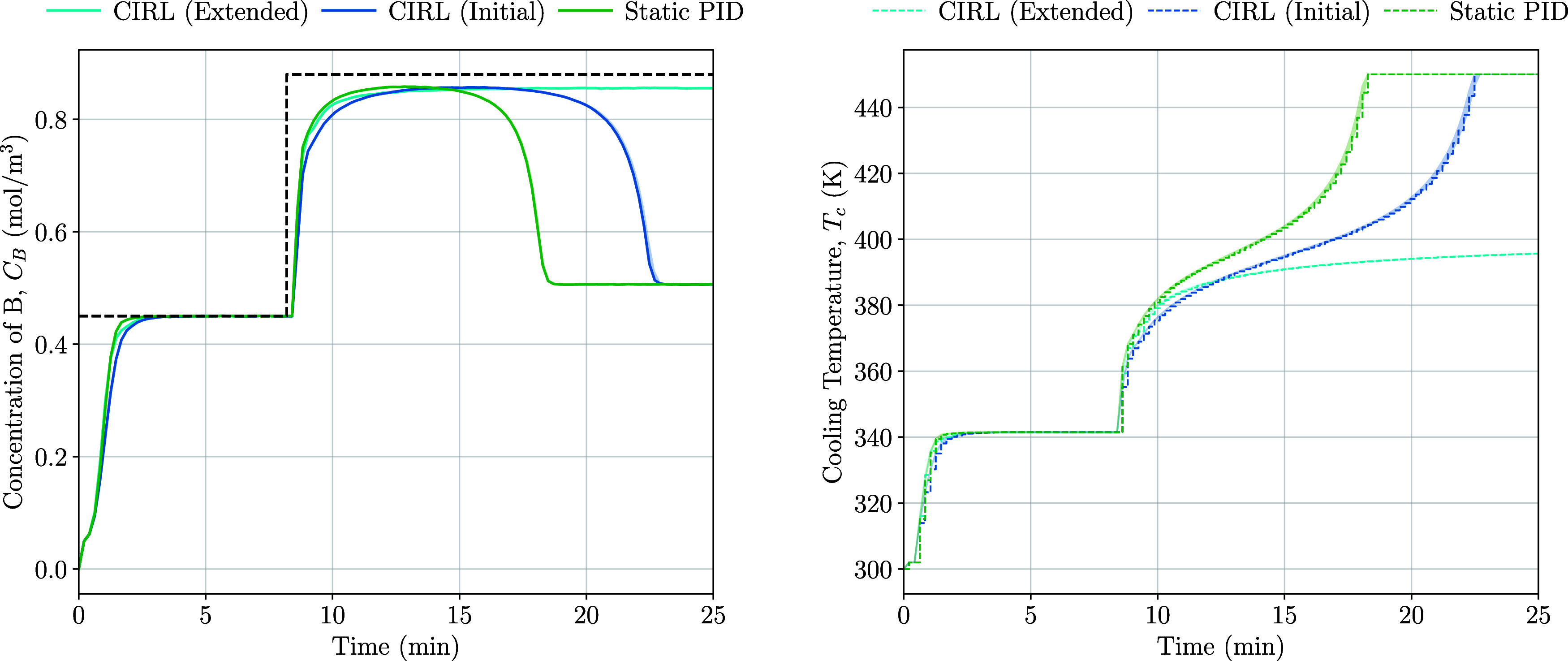
Set point tracking with the static PID control and initial and extended training CIRL.

**Figure 11:**
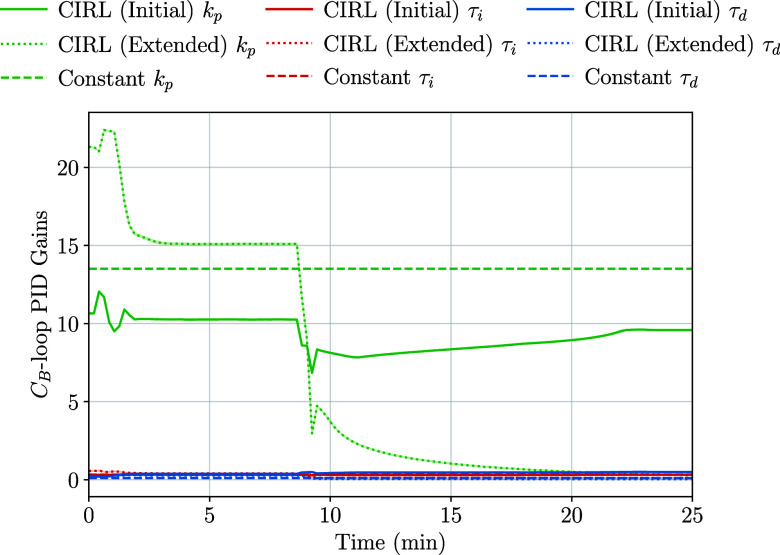
Gain trajectories for the static PID control and initial and extended training CIRL.

This scenario is explored by testing on a new set point schedule for species B of 0.45–0.88 mol/m^3^ (Figure 10). This high operating point scenario shows both the initial CIRL agent, as trained with a schedule shown in Table 3, and the static PID controller, tuned to the extended set point schedule which includes the maximum of the operating region of 0.88 mol/m^3^, enter a closed-loop unstable regime since their gains remain at a large positive value. To attempt to negate this problem, the CIRL agent is trained on an extended training regime that includes the maximum of the operating region. This agent with an extended training regime decreases the proportional gain (CIRL Extended in Figure 11) which stabilizes the response of the controller.

The scenario at high operating points reveals a limitation of the initial CIRL agent, as it enters an unstable closed-loop regime similar to the static PID controller due to the significant changes in gradient at cooling temperatures above 390 K. Nevertheless, the adaptability of the deep RL component of the CIRL framework is demonstrated by extending the training regime to include the upper limits of the operating space, allowing the agent to learn and adjust its control strategy, particularly by reducing the proportional gain, to maintain stability and achieve the desired set point even in the presence of these challenging conditions as shown by the higher test reward in Table 6.

**Table 6:** Final Test Reward for Pure-RL, CIRL, and Static PID for the High Operating Point Scenario (Best Reward Shown in Bold)

method	test reward
CIRL (initial)	–4.04
**CIRL (extended)**	**–2.07**
static PID	–6.81

### 4.6 Disturbance Rejection

We now turn to evaluating the ability of the learned policies to reject disturbances. In particular, the CIRL algorithm is also tested on a scenario where there is a (unmeasured) step change to the feed concentration of species A (*C*_A,in_). Similar to the set point tracking case study, the CIRL and pure-RL algorithms are trained on multiple disturbance subepisodes (Table 7). Then the trained agent is tested only on interpolation within this training regime.

**Table 7:** Training and Test Scenarios

subepisode	disturbance
	*C*_A,in_ [mol/m^3^]
1	1.5
2	1.6
3	1.9
test	1.75

**Figure 12:**
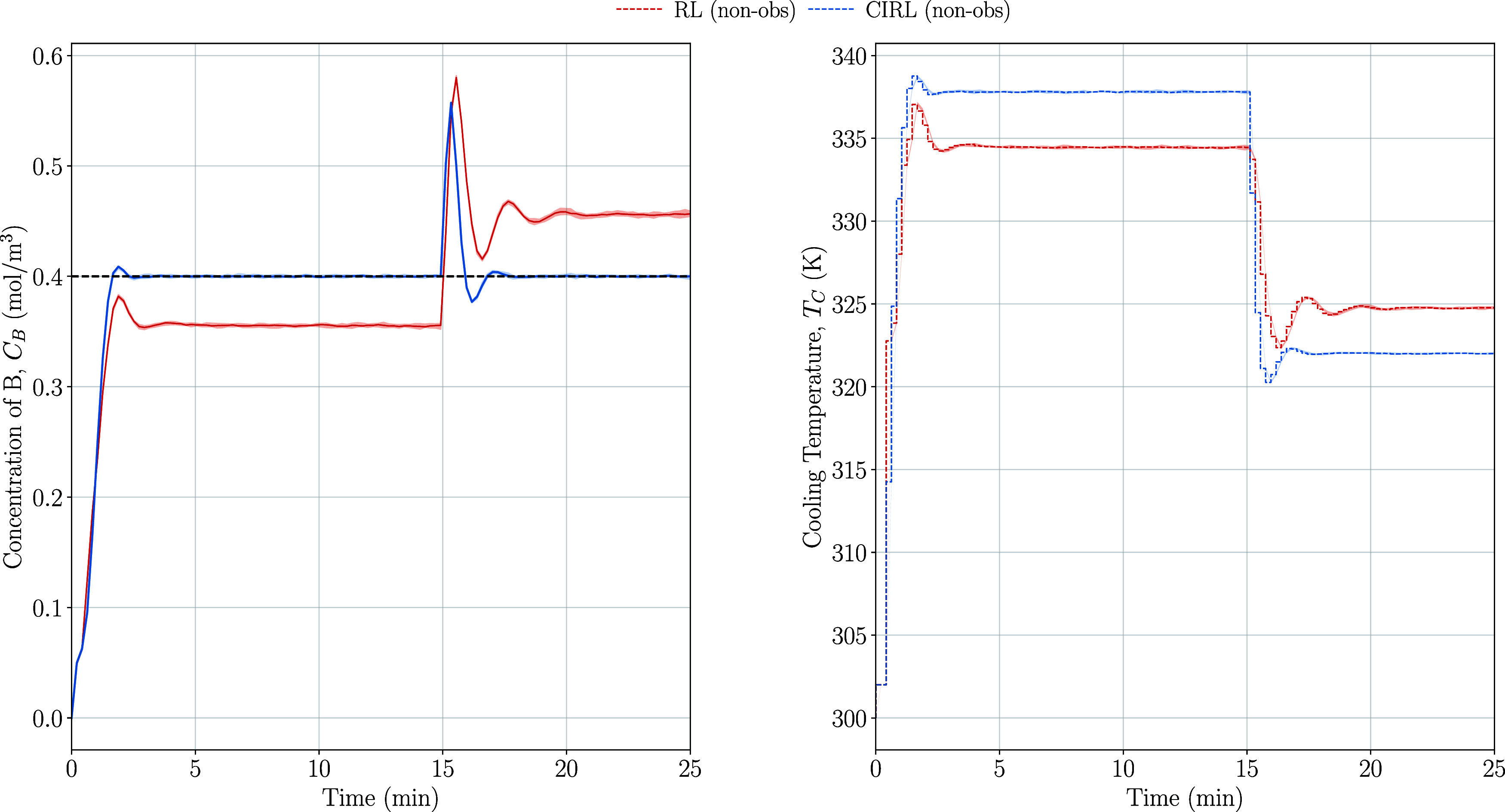
Disturbance rejection test scenario states and control inputs for CIRL and pure-RL with nonobservable disturbance.

Under this disturbance condition, which effectively changes the underlying system dynamics, CIRL demonstrates a good ability to reject the disturbance and maintain the desired set point tracking performance as shown in Figure 12. This disturbance rejection capability stems from CIRL’s integrated PID control structure. The PID components in CIRL continuously measure and respond to the error between the set point and the actual system output, allowing it to adapt to and counteract unexpected disturbances in real time, even if they were not explicitly modeled during training. Conversely, the pure-RL method exhibits poor set point tracking when faced with dynamics outside its training distribution (Table 8). Without an explicit mechanism to handle disturbances, it settles for a compromised policy, i.e., sacrificing set point tracking performance both before and after the test disturbance occurred. This highlights that the addition of the PID components to the CIRL provides robustness to unmodeled disturbances. Unlike the pure-RL approach that attempts to anticipate and learn responses to all possible disturbances during training, CIRL’s PID feedback mechanism allows it to adapt to unforeseen disturbances by using the measured error instead of modeling the response to the disturbance, demonstrating the fundamental advantage of closed-loop control in handling system uncertainties.

**Table 8:** Final Test Reward for Pure-RL, CIRL, and Static PID for Disturbance Rejection

method	test reward
CIRL	–1.38
pure-RL	–1.76

## 5 Conclusions

This work presents a CIRL framework that integrates classical PID control structures into deep RL policies. A case study of a simulated CSTR demonstrates that CIRL outperforms both model-free deep RL and static PID controllers, particularly when tested on dynamics outside the training regime. The key advantage of CIRL lies in the embedded control structure, which allows for greater sample efficiency and generalizability. By incorporating the inductive biases of the PID controller layer, CIRL can learn effective control policies with fewer samples and adapt to novel scenarios more robustly than pure model-free RL approaches.

Future work may seek to incorporate additional existing information regarding the existing PID infrastructure. For example, as a preprocessing step in the algorithm, the neural network could be initialized via offline reinforcement learning or behavioral cloning from past policies, potentially leveraging preexisting gain schedules in the plant. This initialization could potentially improve the starting point for the CIRL framework and accelerate learning. Another direction may be enabling gradient-based training of CIRL agents by investigating the end-to-end differentiability of the PID controller layer. Furthermore, the reward function used in this work is specific to regulatory problems. Different objectives, such as those including process economics, could also be investigated.

The proposed CIRL framework opens up exciting research directions at the intersection of control theory and machine learning. This combination seeks to benefit from the best of both worlds, merging the known disturbance-rejection and set point-tracking capabilities of PID control with the generalization abilities of machine learning. Further investigations into theoretical guarantees and online adaptation schemes have the potential to enhance the sample efficiency, generalization, and real-world deployability of deep RL algorithms for control applications across various industries. The code and data used within this work are available at https://github.com/OptiMaL-PSE-Lab/CIRL.

## RGA Matrix

The RGA matrix used for controller pairing is displayed below using averaged system gains over three repetitions of an applied step change:

20

The values in the RGA matrix suggest a strong pairing between the first controlled variable (*C*_B_, the concentration of B) and the second manipulated variable (*T*_c_, the cooling temperature) and between the second controlled variable (*V*, the volume) and the first manipulated variable (*F*_in_, the inlet flow rate).
